# Sintilimab combined with AVD for the treatment of composite Hodgkin lymphoma and follicular lymphoma: a case report and literature review

**DOI:** 10.3389/fonc.2025.1692954

**Published:** 2026-01-12

**Authors:** Huami Ye, Linlin Huang, Changqian Chen, Shihua Huang, Lianjie Hu, Ling Wang

**Affiliations:** 1 Department of Hematology, The Second People’s Hospital of Yibin, Yibin, Sichuan, China; 2 Pathological Diagnosis Center, Sichuan Kingmed Center for Clinical Laboratory CO, Ltd, Chengdu, Sichuan, China

**Keywords:** composite lymphoma, Hodgkin lymphoma, follicular lymphoma, PD-1 inhibitors, treatment

## Abstract

Composite lymphoma has been rarely reported due to its low incidence. The treatment of composite lymphoma depends on the specific lymphoma subtypes involved. No standardized therapeutic regimen has been established. We tried to treat the patient with Sintilimab+AVD for composite Hodgkin lymphoma and follicular lymphoma, after which the patient was able to maintain a normal quality of life and achieved partial remission (PR). To the best of our knowledge, this is the first attempt to use the PD-1 inhibitor combined with the AVD regimen successfully for the treatment of composite Hodgkin lymphoma and follicular lymphoma.

## 1 Introduction

Classical Hodgkin lymphoma (cHL) usually has an aggressive clinical presentation, AVD+PD-1 or BV+AVD has already been widely applied in cHL, and BV-AVD is more toxic than ABVD in adults (1). PD-1 blockade is a safe and effective treatment; high response rates and durable remissions are observed following PD-1 blockade in untreated cHL than BV (2–7).

Follicular lymphoma (FL) is the most common indolent lymphoma worldwide (8), FL grade 3 (FL3) commonly leads to worse overall survival (9, 10), and BR or R-CHOP regimens are effective options for patients with grade 3a FL (11). In relapsed/refractory follicular lymphoma, the objective response rate of PD-1 inhibitor monotherapy is 40% (12).

A composite lymphoma is defined by the presence of at least two lymphomas in the same anatomical site (13). Composite lymphoma is a rare entity. To date, only a limited number of case reports have described therapeutic approaches for composite Hodgkin and follicular lymphoma (14, 15). It seems impossible to conduct large prospective clinical trials in composite lymphoma due to its low incidence. Therefore, the best treatment option for composite Hodgkin lymphoma and follicular lymphoma has not been found. There are currently no available data on the use of the PD-1 inhibitor combined with the AVD regimen in patients with Hodgkin follicular composite lymphoma. In order to enrich the experience in the treatment of cHL/follicular composite lymphoma, we report a case of Hodgkin follicular composite lymphoma on Sintilimab+AVD regimen, through which the patient achieved cancer remission and a longer survival duration.

## 2 Case presentation

### 2.1 Initial diagnostic-therapeutic phase

A 50-year-old woman presented to an outside hospital in December 2023 with >2 years of recurrent cough, expectoration, and cervical lymphadenopathy. Blood work showed chronic anemia, with hemoglobin levels ranging from 57 to 70 g/L.PET/CT imaging demonstrated the following findings: 1) lymphoma involvement of multiple lymph nodes throughout the body; 2) diffuse increased glucose metabolism in the bone marrow, with lymphoma invasion not excluded; 3) lesions in the greater omentum and small intestinal mesentery, most consistent with panniculitis; 4) splenomegaly and scattered ascites in the abdominal cavity; 5) bilateral pulmonary lesions, suggestive of inflammation. The pathological report provided by the patient’s family indicated the following: the left cervical lymph node biopsy was consistent with a lymphoid tissue neoplasm, and differential diagnosis was required between peripheral T-cell lymphoma (with HRS cells) and classical Hodgkin lymphoma (cHL). However, the original report form is not available. The external hospital diagnosis was peripheral T-cell lymphoma (IIIB). The patient completed five cycles of chidamide + CHOP at an outside hospital. Post-treatment follow-up PET/CT revealed new lymphadenopathy, progression of existing nodal lesions, and the unavailability of the original PET/CT report for comparison. The patient discontinued treatment.

### 2.2 Second diagnostic-therapeutic stage

#### 2.2.1 Medical history overview

The patient was emergently admitted to our department in November 2024 due to clinical deterioration, presenting with cough, expectoration, and fever. Physical examination findings: T39.0°C, P135 beats/min, R30breaths/min, BP73/35 mmHg, SpO_2_88%; severe anemia, cachectic appearance, generalized superficial lymphadenopathy, decreased breath sounds in bilateral lower lung fields, non-palpable hepatosplenomegaly, and extensive generalized edema.

#### 2.2.2 Hematological and bone marrow examination results

Peripheral blood analysis

White blood cells (WBC): 20.64×10^9^/L

Neutrophils: 19.4×10^9^/L

Lymphocytes: 0.76×10^9^/L

Red blood cells (RBC): 0.75×10¹²/L

Hemoglobin (Hb): 18 g/L

Platelets: 188×10^9^/L

Reticulocytes: 22.91×10^9^/L

Procalcitonin (PCT): 6.04 ng/ml

Bone marrow examination

Cytology: markedly active granulocytic hyperplasia, with erythroid proliferation reduced to 6% of the total nucleated cell count.

Flow cytometry: no significant abnormalities detected.

#### 2.2.3 Diagnostic and therapeutic management

**Figure 1:**
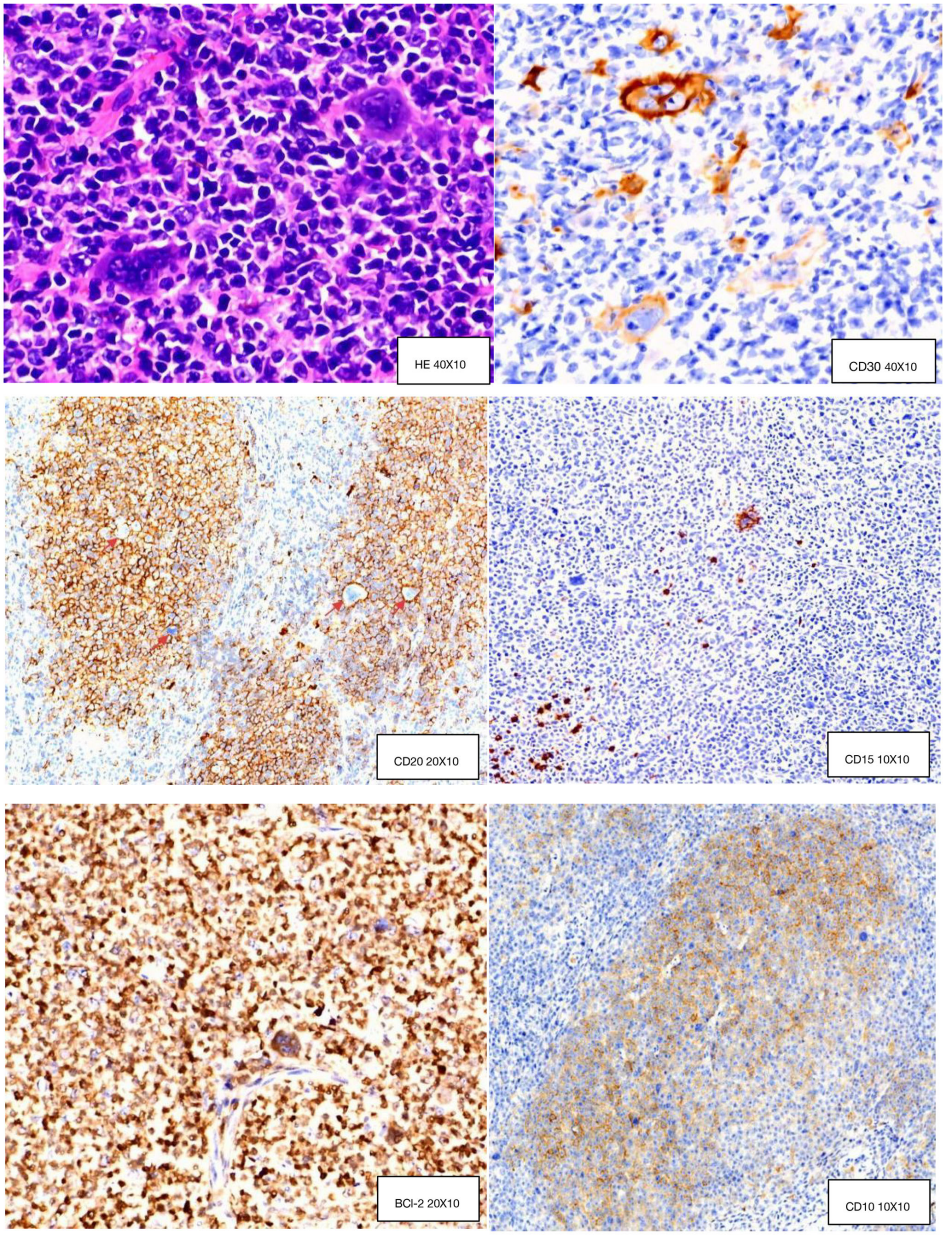
Hematoxylin and eosin (H&E) staining and immunohistochemistry (IHC) analysis.

The patient received immediate symptomatic management including antimicrobials, fluid resuscitation, anti-hypotensive therapy, and blood transfusion. Given the patient’s critical condition, lack of response to the CHOP-like regimen, and the need to differentiate Hodgkin lymphoma (per external pathology), the ABVD regimen was initiated on November 24, 2024, for one cycle. After treatment, the patient’s fever and productive cough gradually improved; however, generalized superficial lymphadenopathy persisted. Left inguinal lymph node biopsy was done on December 30, 2024. Histopathology: composite lymphoma (cHL+FL3a). IHC: CD20(+), CD19(+), LCA(+), CD10(+), bcl-2(+), bcl-6(+),CD30(+), MUM-1(−), Ki67(+, 80%), CD3(−), CD5(−),CyclinD1(−),CD15(−),CD21(+) (FDC network); background T cells: CD3(+),CD5(+),PD-1(+)(**Figure 1**). EBER1/2 ISH: negative. Gene rearrangement: IgH and IgK clonal amplification, no TCRG clonal rearrangement.

**Figure 2:**
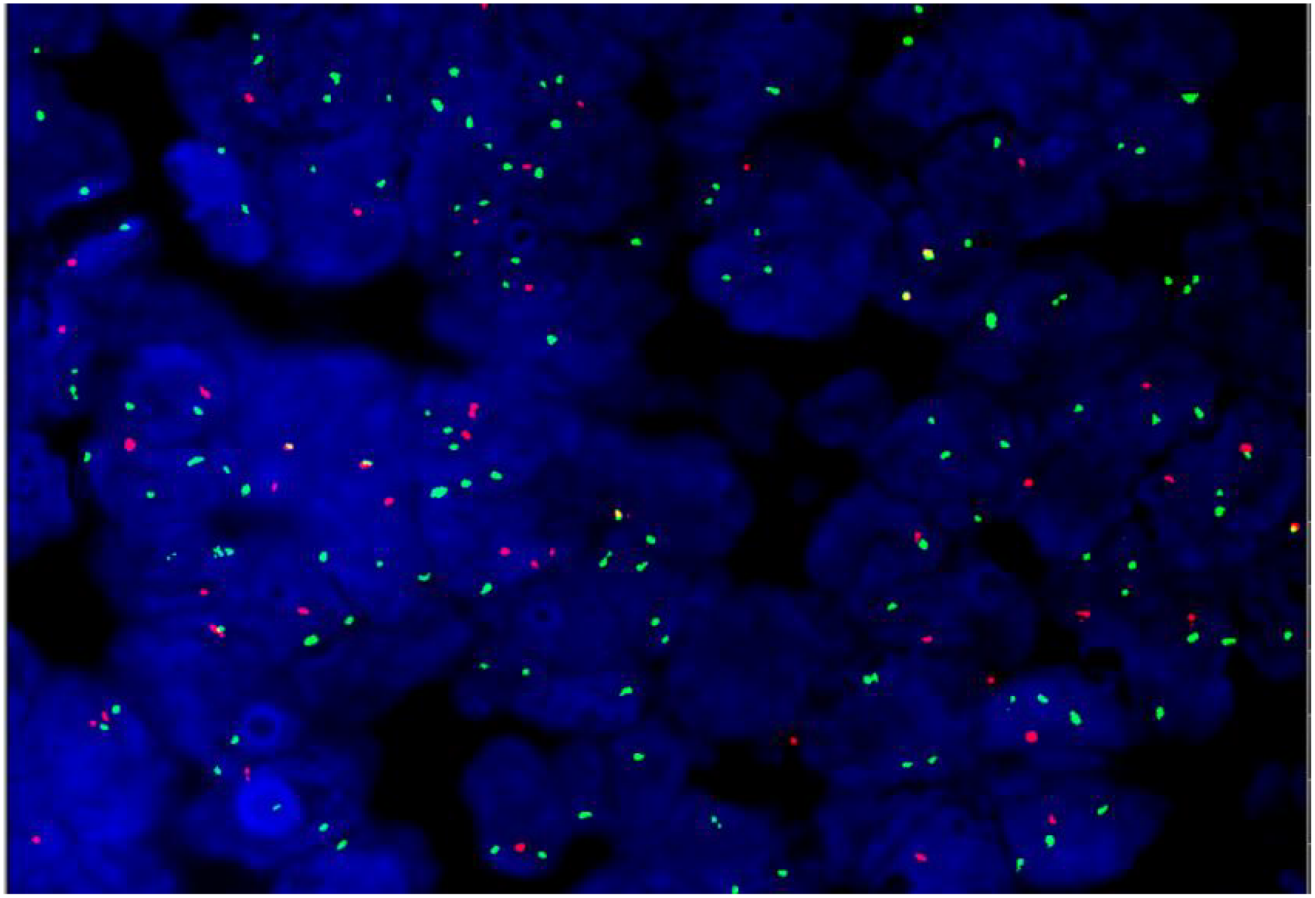
Bcl-2 positive.

FISH analysis demonstrated positivity for the BCL-2 gene (**Figure 2**), with no abnormalities detected in the BCL-6 or MYC genes.

Pathological tissue mutation profiling revealed the following genetic alterations: *TNFRSF14, KMT2D* (class I), *CBLB, TNFAIP3, TP53* (class II), *TBL1XR1, FAT4, SETD1B, RB1, and BCORL10* (class III). This case also showed multiple chromosomal or chromosomal segmental abnormalities, including those in *chr1, chr3, chr4q, chr6, chr9p, chr17, and chrX.*

**Figure 3:**
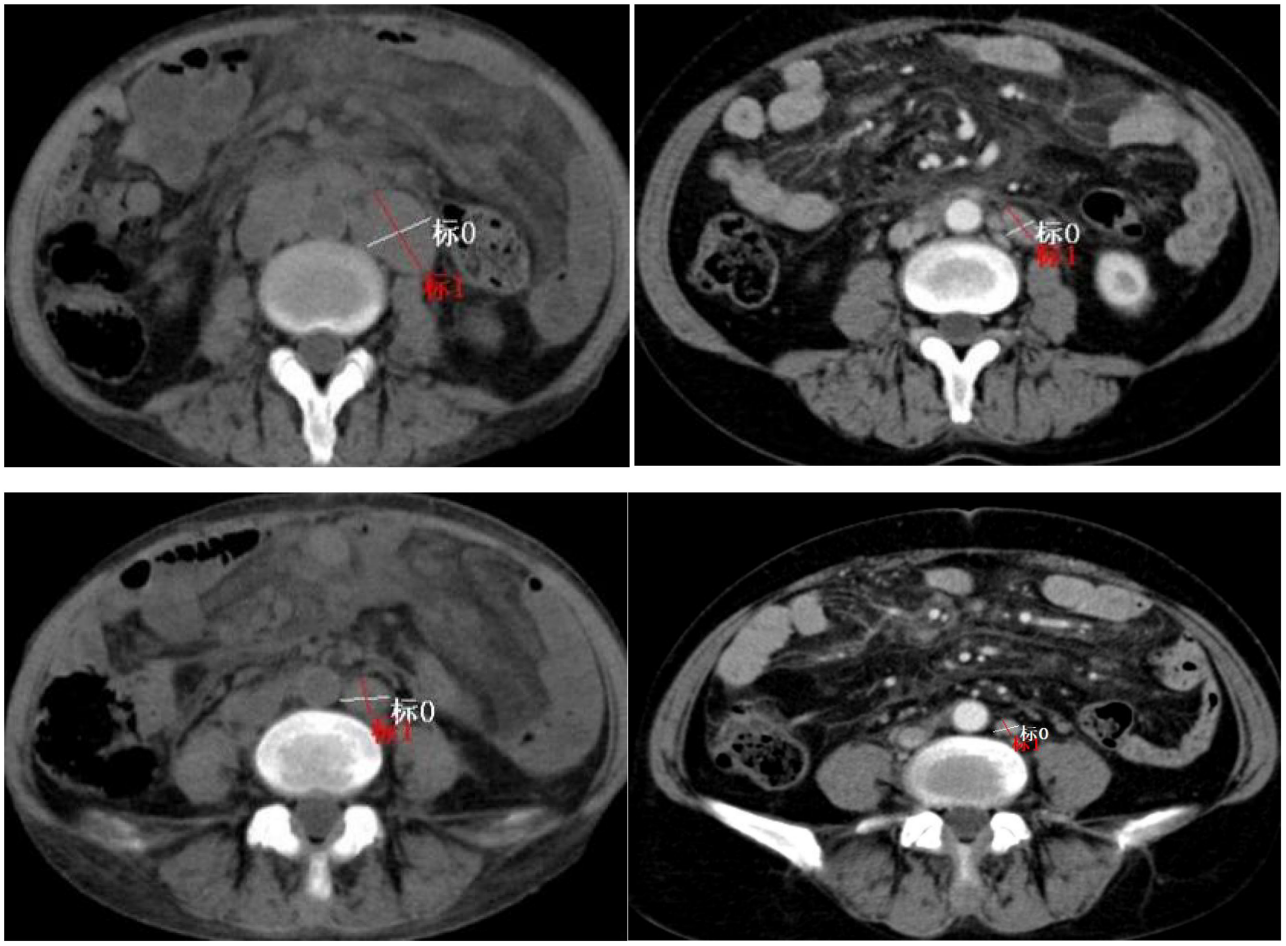
CT scan prior to initiation of PD-1 inhibitor treatment (12 Dec. 2024) (left), and six cycles after treatment initiation (3 Jun. 2025) (right).

The patient responded well to the first ABVD cycle. Inguinal lymph node pathology identified composite classical Hodgkin lymphoma grade 3a follicular lymphoma, leading to treatment switch to Sintilimab-AVD (Sintilimab 200 mg q21d; vincristine 2 mg, doxorubicin 25 mg/m², dacarbazine 375 mg/m² days 1/15) for 5 cycles. Financial constraints precluded PET/CT; contrast-enhanced CT showed 60.78% target lymph node reduction *vs*. baseline (PR; **Figure 3**), ECOG PS 0. Due to chemo cytotoxicity and prior treatment, combination therapy was stopped in June 2025, and Sintilimab maintenance (200 mg q21d) was initiated. Hemoglobin normalized; follow-up will continue. No grade 3/4 treatment toxicities or immune-related adverse events were noted.

## 3 Discussion

### 3.1 Diagnostic evaluation and interpretation

Hodgkin and Reed–Sternberg (H-RS)-like cells can occur in T-cell non-Hodgkin lymphoma (16); thus, the external hospital’s pathology department misdiagnosed this case as peripheral T-cell lymphoma with Reed–Sternberg (RS) cells. We analyzed this composite lymphoma case, where both follicular lymphoma (FL) and classical Hodgkin lymphoma (cHL) involved the same lymph node—both fulfilling the required diagnostic immunohistochemical (IHC) features. Fluorescence *in situ* hybridization (FISH) showed BCL-2 positivity, and gene rearrangement studies detected clonal amplification peaks of IgH and IgK. Combining the patient’s pathological findings, IHC results, and genetic data, the definitive diagnosis is composite lymphoma consisting of FL and cHL.

### 3.2 Therapeutic management and clinical decision-making

Combination therapeutic regimens for two types of aggressive lymphoma are exceedingly rare. Alexis Trecourt (17) analyzed 40 cases of composite lymphoma: 10 received cHL-like chemotherapy, 12 small/diffuse B-cell lymphoma-like chemotherapy, 14 composite lymphoma-like chemotherapy, 1 radiotherapy, and 3 no treatment. Approximately 50% of patients in each of the three chemotherapy groups remained alive in remission, with no mention of risk stratification for any patient. Our literature review found two case reports describing two patients with transformed or composite lymphoma who achieved favorable efficacy and good safety with PD-1 inhibitors after multiple lines of treatment (18, 19). Another case report described a patient with transformed follicular lymphoma (tFL) who was unresponsive to CAR-T therapy but achieved complete remission (CR) with PD-1 inhibitor plus radiotherapy (20).

This patient had previously failed the CHOP regimen and harbored complex karyotypes, relevant gene mutations, and acquired *TNFRSF14* mutations—a marker linked to worse prognosis in FL and reduced benefit from rituximab (21). Notably, *TNFRSF14* and *KMT2D* mutations may drive FL transformation to diffuse large B-cell lymphoma (22). The patient also carried truncating mutations in *CBLB* and *TNFAIP3*, and a missense mutation in *TP53*; all these tumor-suppressor genes, when mutated, can inactivate protein function and potentially promote tumor initiation and transformation.

Due to the patient’s CHOP failure and high-risk composite lymphoma status, plus poor general condition (which made rituximab-based intensive therapy unlikely beneficial), PD-1+AVD was selected. This choice was based on literature analysis and the patient’s favorable response to prior ABVD: PD-1 targets both Hodgkin’s lymphoma (HL) and FL, and FL’s dual aggressive-indolent traits make it hard to control with chemotherapy alone (supporting PD-1 maintenance therapy).

This is the first reported case of the PD-1 inhibitor combined with AVD as first-line therapy for composite classical HL (cHL) and FL, with good treatment tolerance.

### 3.3 PD-inhibitor mechanisms in composite lymphoma

Consensus exists on the PD-1 inhibitor use for Hodgkin’s lymphoma (HL), with studies also exploring its application in follicular lymphoma (FL). Programmed death 1 (PD-1), a member of the CD28/CTLA-4 family, negatively regulates T-cell receptor signaling and reduces T-cell proliferation and cytokine production. PD-1 and its ligands (PD-L1, PD-L2) are widely expressed in tumor microenvironments—including in epithelial malignancies, classical HL, and non-Hodgkin lymphoma (NHL)—with more evidence supporting PD-L1’s role in FL (23). PD-L1 binding to PD-1 on tumor-infiltrating lymphocytes impairs T-cell proliferation and cytotoxicity (12), whereas monoclonal antibody blockade of the PD-1/PD-L1 axis restores cytotoxic T-cell antitumor activity (13). Numerous studies confirm BCL-2 co-expression and clonal IGH/IGK rearrangements in the histiocytes of both HL and FL. These shared genetic alterations have led to the hypothesis that the two lymphoma subtypes share a common cellular origin (15, 24–26). Given the patient’s pathological findings—background T-cell PD-1 (+), Bcl-2 (+), and detected IgH/IgK rearrangement—we hypothesize that composite follicular lymphoma (FL) and classical Hodgkin lymphoma (cHL) share the same tumor microenvironment. This supports the notion that Sintilimab+AVD combination therapy, along with Sintilimab maintenance therapy, yields favorable outcomes in composite cHL+FL. The mechanism of action of PD-1 inhibitors on the tumor microenvironment in composite lymphoma requires further confirmation.

## 4 Conclusion

To our knowledge, this is the first report of the PD-1 inhibitor combined with the AVD regimen for composite Hodgkin’s lymphoma (cHL) and follicular lymphoma (FL) and it achieved favorable clinical outcomes.

The tumor microenvironment of cHL is characterized by high PD-L1 expression, whereas PD-1 inhibition may also counteract immune evasion in FL through synergistic effects. This supports the dual therapeutic potential of immunotherapy for composite lymphomas.

Sequential PD-1 inhibitor + AVD followed by PD-1 maintenance therapy may thus be a viable systemic strategy for patients with composite cHL and grade 3a FL. However, this study only included one case, so additional cases and longer follow-up are needed for further validation.

## Data Availability

The original contributions presented in the study are included in the article/supplementary material. Further inquiries can be directed to the corresponding author.
